# Gluten-Free Diet in Celiac Disease—Forever and for All?

**DOI:** 10.3390/nu10111796

**Published:** 2018-11-18

**Authors:** Alice Itzlinger, Federica Branchi, Luca Elli, Michael Schumann

**Affiliations:** 1 Division of Gastroenterology, Infectious Diseases and Rheumatology, Campus Benjamin Franklin, Charité Universitätsmedizin Berlin, 12203 Berlin, Germany; alice.itzlinger@charite.de (A.I.); federica.branchi@charite.de (F.B.); 2 Center for the Prevention and Diagnosis of Celiac Disease, Fondazione IRCCS Ca’ Granda Ospedale Maggiore Policlinico, 20122 Milan, Italy; luca.elli@policlinico.mi.it

**Keywords:** gluten, adherence, serology, villous atrophy, mucosal recovery, threshold

## Abstract

The gluten-free diet is the only effective treatment available for celiac disease. However, it is difficult to adhere to and a closer look on the diet’s implementation and indications reveals several ambiguities: Not only is there controversy on the threshold of gluten that can be tolerated in the frame of a strict gluten-free diet, but it is also unclear whether the gluten-free diet is an appropriate treatment in patient subgroups with asymptomatic or potential celiac disease. Reports from a number of research groups suggest that a certain proportion of patients may effectively develop tolerance to gluten and thus become suitable for gluten reintroduction over time. In this review, we set out to create an overview about the current state of research as regards the definition of a strict gluten-free diet in terms of the gluten thresholds considered tolerable and the indication for a gluten-free diet in the absence of histological abnormalities or symptoms. Furthermore, we discuss the concept that a gluten-free diet must be followed for life by all patients.

## 1. Introduction

Celiac disease (CD) is an autoimmune disorder triggered by the ingestion of gluten. As a consequence of dietary exposure to gluten, genetically susceptible individuals develop autoimmune reactions resulting in histological changes in the small intestine. These are characterized by duodenal villous atrophy and intraepithelial lymphocytosis (IEL), leading to malabsorption and gastrointestinal as well as extraintestinal symptoms [1]. The clinical presentation of CD is diverse, with a considerable number of patients being asymptomatic. The classical form of CD is characterized by an overt malabsorption syndrome that features chronic diarrhea, steatorrhea, weight loss, and failure to thrive in children. Gastrointestinal symptoms as well as extraintestinal symptoms such as fatigue, osteopenia, iron deficiency, anemia and neurological/psychological disorders such as depression may be present [1,2]. On the other hand, in the non-classical form of CD patients present only one or few gastrointestinal or extraintestinal symptoms [2]. Table 1 shows the characteristics of different clinical presentations.

A lifelong gluten-free diet (GFD) has long been considered the only effective treatment for CD. Strict adherence to a GFD has been shown to lead to partial—if not complete—healing of the duodenal mucosa along with resolution of symptoms and signs of malabsorption [2]. It has been observed that mucosal recovery takes more time and is more frequently incomplete in adults than in children [3,4]. There remain, however, open issues regarding not only the tolerable threshold of gluten ingestion but also the appropriateness of a lifelong indication to GFD, particularly in patients with subclinical and potential CD. Considering the impact on the patients’ quality of life posed by a restrictive GFD [5,6], its necessity should be reviewed carefully when prescribing it.

First of all, the natural course of CD is not as clearly understood as one might think. It is still unclear if a strict GFD needs to be followed throughout the whole life in all patients or if tolerance may develop in certain patients. While the GFD generally leads to clinical improvement, mucosal abnormalities may persist, but do not add up to functional malabsorption as clinical symptoms decline. Rubio-Tapia et al. observed mucosal recovery in about 35% two years after starting a GFD and in about 66% after five years; however, 82% improved clinically. In line with these findings, clinical improvement of symptoms was not a reliable marker of mucosal recovery (*p* = 0.7) [7]. On the other hand, cases of patients who—in many cases on their own—interrupt the GFD and remain symptom-free have been reported in some studies [8].

Furthermore, while there are strong data supporting the benefits of a GFD in patients with symptomatic CD or CD irrespective of symptomatology, the outcome of GFD within the subgroups of asymptomatic and potential CD is mostly unclear. In these patients, the response to GFD can neither be measured in terms of symptom relief—nor in terms of mucosal healing for potential CD. For this reason, the issue of the actual role of the GFD in the prevention of CD-related complications (e.g., refractory disease, intestinal lymphoma) and other immune disorders should be addressed. In view of the scattered data on the risk of CD-related complications in these patient groups, the prescription of a GFD needs to be critically debated.

The aim of this article is to critically review existing literature data on the therapeutic and prognostic role of the GFD in CD in order to give an overview about the current clinical indications for the GFD and future perspectives for celiac patients.

## 2. Methods

A comprehensive literature search was carried out on PubMed to identify peer-reviewed articles on GFD in CD published until August 2018. Search strategies included the following key words: celiac disease, gluten-free diet, adherence, autoimmune disorders, gluten re-challenge, gluten tolerance, gluten contamination, celiac disease complications, lymphoproliferative disorders, and enteropathy-associated T-cell lymphoma. A manual search was also carried out on the bibliographies of the identified articles. The most relevant original research articles in the English language, including randomized controlled trials and observational studies (prospective and retrospective) were selected by the authors and are discussed in the following sections.

## 3. Gluten-Free Diet: How Strict Should It Be?

Current guidelines suggest that the GFD should be strict, with complete avoidance of gluten containing products and attention to cross-contaminations [2].

Gluten contamination, even within the frame of a strict GFD, cannot be entirely avoided: Plenty of products contain hidden gluten, such as sausages, soups, soy sauce and ice cream. But even in gluten-free-labeled products there are traces of gluten. This is mostly due to cross-contamination with gluten-containing products that are processed or stored in the same place [9]. The term “gluten-free” thus generally refers to an amount of gluten that is thought to be harmless and does not imply total absence of gluten. In fact, the amount of gluten deriving solely from cross-contamination in a supposedly GFD may range from 5 to 50 mg per day [10,11]. The level of gluten content in food products is expressed as parts per million (ppm, corresponding to mg/kg). Gibert et al., collected consumption data of gluten-free products by CD patients in order to estimate the average gluten exposure of celiac patients who follow a GFD. Taking into account different dietary habits in European countries (Norway, Germany, Italy, Spain) they concluded that a limit of 20 ppm for products naturally gluten-free and 100 ppm for products rendered gluten-free would be acceptable [12]. According to the current guidelines of the European Commission a commercially sold product may be called “gluten-free” if it contains less than 20 ppm gluten (20mg/kg) [13].

Several studies have tried to establish a safe threshold of daily gluten intake, as summarized in Table 2. A prospective, multicenter, placebo-controlled, double-blind, randomized gluten challenge trial by Catassi et al., found that the chronic ingestion of small amounts of gliadin leads to a dose-dependent relapse in symptoms: children diagnosed with CD who had followed a GFD for at least three months were given small amounts of gluten for a period of four weeks [14]. Patients who had received 100 mg gliadin/day (= about 200 mg of gluten or the equivalent of 2–5 g wheat flour) displayed minimal morphometric changes in the jejunal histology. Patients who had ingested 500 mg gliadin per day showed significantly more histological abnormalities. Additionally, some patients became positive to antibodies again and experienced a symptom relapse. These results support the findings of a previous study by Ciclitira et al., who found no changes in mucosal histology after the infusion of 10 mg gliadin, minimal changes after 100 mg of gliadin and significant changes after an additional 500 mg of gliadin [15].

A further prospective, double blind, placebo-controlled trial by Catassi et al., analyzed the effects of daily ingestion of 0, 10, or 50 mg gluten for 90 days [9]. While the villous height to crypt depth ratio of the placebo group raised by 9%, no difference was found in the 10 mg group and a −20% was found in the group receiving 50 mg gluten/day. The IEL count did not differ significantly between the three groups. Catassi et al., concluded that the ingestion of contaminating gluten should be kept lower than 50 mg/day in the treatment of CD [9,16].

Two systematic reviews aimed to determine the safe maximum gluten intake. Hischenhuber et al., suggested that the maximum daily gluten intake should lie between 10 and 100 mg [11]. Akobeng and Thomas included thirteen studies and similarly found that the amount of tolerable gluten varied widely between the studies, ranging from patients who tolerated an average of 34–36 mg gluten per day to others who developed symptoms and/or histological abnormalities when consuming 10 mg gluten per day [17]. The results of these systematic reviews show that there is no single definitive threshold for gluten intake but that a daily intake of <10 mg is unlikely to cause mucosal abnormalities (see also Table 2).

### More Than A Strict Gluten-Free Diet

Several research groups have observed that in a considerable portion of patients complying to a presumed strict GFD, complete mucosal recovery and/or resolution of symptom do not occur [5,19,20,21,22]. These results led to questions regarding the efficacy of the GFD in obtaining histological remission.

The first step in patients with signs of non-responsive CD should be the evaluation of the GFD by a trained dietitian to unveil possible unwitting intake of gluten. However, current methods and questionnaires still rely on the patients’ subjective assertions and not able to accurately measure dietary compliance [23]. A relatively new method to monitor GFD compliance is the detection of gluten immunogenic peptides that can be found in the urine and feces after gluten consumption. This offers an objective method to assess the dietary adherence and has shown to be very sensitive [24,25,26].

While most patients tolerate trace gluten under the 20 ppm threshold, a subgroup might react even more sensitively. It has therefore been suggested that a more-than-strict GFD regimen has the potential to induce mucosal recovery in these patients [27]. The Gluten Contamination Elimination Diet (GCED) is a diet that was developed to remove even minute amount of gluten from the diet, consisting of a more restrictive dietary regimen focusing on the use of naturally gluten-free products rather than processed gluten-free food [27,28,29].

Hollon et al., identified 17 patients not responsive to GFD. Before starting the GCED, all subjects were assessed by a dietitian who excluded hidden gluten ingestion. After completing the GCED for 3–6 months, 82% responded to the GCED and became asymptomatic [28].

Zanini et al., investigated if complete mucosal recovery can be achieved by adopting this restrictive diet or, alternatively, after a prolonged time of GFD [29]. In this small study, after three months of GCED, no changes in villous height and crypt depth were observed as compared to two years of GFD. Only a slight, though significant decrease in IELs and T-cells with a distinctive T-cell receptor (TCR) on their surface, so called TCRγδ+ cells, was found. The study suggested that the intraepithelial lymphocytosis persisting in CD patients during GFD cannot completely be eliminated even on a GCED and is furthermore independent of the duration of adherence to a standard GFD. Whether gluten contamination is unavoidable despite GCED, or a three-month period of GCED was too short, could not be clarified by the study, possibly also due to the small sample size [29]. Given the scarce data, there is insufficient evidence to recommend a gluten contamination elimination diet on a routine basis in patients with CD. However, it should be considered in patients who are not responsive to traditional GFD.

## 4. Does the Gluten-free Diet Prevent the Development of Autoimmune Diseases and of Celiac Disease Complications?

### 4.1. Diet and Autoimmune Diseases

Patients with CD are at increased risk for various autoimmune disorders, particularly diabetes mellitus type I and autoimmune thyroiditis but also autoimmune hepatitis, rheumatoid arthritis and Sjögren’s syndrome among others [30,31,32]. The same applies vice versa: Patients with autoimmune diseases have a significantly higher prevalence of CD than the healthy population—a Norwegian study found that around 5% patients with type 1 diabetes also have celiac disease [30]. The association between CD and other autoimmune conditions is presumably caused by a common genetic background (the presence of predisposing human leucocyte antigen (HLA) class II haplotypes), but the direct role of gluten exposure in the development of associated autoimmune conditions is still a matter of debate [33].

There are a number of contradictory studies concerning the association between the development of an additional autoimmune disorder and the duration of gluten exposure. Initially, it was hypothesized that strict adherence to GFD could prevent the development of further autoimmune diseases in patients with celiac disease. A cross-sectional study by Ventura et al., in 1999 showed an association between the prevalence of autoimmune disorders in CD patients and the age at diagnosis of CD, suggesting that duration of exposure to gluten might be a predictor of the development of other autoimmune conditions [34]. However, more recent studies tend to argue against a connection: A study by Sategna et al., found no correlation between the duration of exposure to gluten and the prevalence of autoimmune diseases [35]. Similar results were shown in a Finnish study in 2005 [31] as well as by a follow-up study by the same group [36].

All in all, current data allow for the assumption that the GFD has no definite role in the prevention of development of other autoimmune conditions.

### 4.2. Diet and Celiac Disease Complications

In children, early detection and treatment of celiac disease are of utmost importance to prevent complications such as poor growth, decreased bone mineral density, and enamel defects which can be irreversible [37,38]. But even for adults, a diagnostic delay is associated with decreased quality of life, more days of sickness, and frequent use of medicines and health care services [39]. The GFD is an effective tool against gastrointestinal and extraintestinal manifestations as well as complications such as malabsorption and osteoporosis [39,40,41].

Another complication is refractory CD which is defined as the persistence of clinical signs of CD, as well as villous atrophy, despite adherence to a strict GFD for at least 12 months [1]. Of the two subtypes of refractory CD, type II is associated with a worse prognosis in view of the high risk of development of malignancy. The enteropathy-associated T-cell lymphoma (EATL) is a lymphoproliferative disorder that typically develops in the setting of refractory CD. This is a rare type of T-cell lymphoma that arises from intraepithelial T-cells in the small intestine. A higher risk for developing other malignancies, including small bowel adenocarcinoma, has been reported in the setting of CD, but the underlying pathogenesis is still unclear [42,43,44,45,46].

Given the poor prognosis and limited therapeutic options available for type II refractory CD and EATL, research focused on both early diagnosis and identification of risk factors. The role of the GFD for the prevention of EATL development is controversial. From a pathophysiological point of view, EATL develops from aberrant intestinal lymphocytes that proliferate clonally independently of gluten exposure—a longer exposure to gluten, and thus a prolonged immune activation in the intestinal mucosa, may in fact contribute to create a favorable environment to the development and proliferation of aberrant lymphocytes [47]. However, the natural history of CD and the actual prognostic effect of the GFD are difficult to investigate and mainly based on retrospective data analysis.

Observational studies investigating the association between undiagnosed CD and mortality risk showed contradicting results, possibly also due to difficulty of differentiating between undiagnosed asymptomatic CD from mono-/oligosymptomatic or symptomatic CD based on medical record and patients reports. Rubio-Tapia et al., reported an up to 4-fold increased mortality rate in undiagnosed CD during 45 years of follow up, suggesting both a possible role of gluten exposure in the development of malignancies and the necessity of very long follow-ups in order to investigate this area [42]. Similarly, a German study showed an excess in mortality, particularly due to cancer, in patients with elevated transglutaminase antibody levels [43]. On the other hand, population-based study like that by Lohi et al., could not find an excess of mortality in unrecognized CD, though reporting a tendency to die from lymphoproliferative disorders, stroke and respiratory diseases [48]. In a case-control study published in 2011, Olén et al., assessed the characteristics of CD patients with particular attention to their diet compliance and compared it with the risk of developing malignant lymphoma [49]. They could not find an association between the development of lymphoma and non-compliance to GFD even though they could not entirely exclude a moderate effect. A study by Biagi et al., suggested the amount of exposure to gluten to be a relevant risk factor for developing malignancies such as EATL [50]. This hypothesis was based on an indirect link: the authors observed that reported mortality rates in CD patients were higher in Southern than in Northern Europe and correlated with the gluten content of the typical diet of each country. Hence, it was presumed that the amount of dietary gluten ingested (before and after diagnosis) was responsible for a higher incidence of complications and higher mortality.

Given the ambiguity that exists, we cannot exclude a causal association between duration and amount of gluten consumption and the development of refractory celiac disease (RCD) and malignancies. It therefore remains essential to advise and stress the importance of a strict GFD to patients with celiac disease. Further research and in particular prospective studies about the aforementioned linkage are needed in order to clarify the role of gluten exposure in this setting.

## 5. Gluten-Free Diet in Potential Celiac Disease

Potential CD is characterized by positive endomysium and/or anti-transglutaminase antibodies and a normal intestinal histology [1]. It is important to emphasize the distinct difference between potential and asymptomatic celiac disease. While all other forms of celiac disease are characterized by intestinal mucosal abnormalities, patients with potential celiac disease do, by definition, not display any histological abnormalities. Individuals with potential CD are often identified as a result of screening or incidental findings on routine examinations (e.g., screening of first-degree relatives or after diagnosis of related autoimmune diseases). The question arises whether individuals with potential CD benefit from a GFD or if the GFD results in a social and economic burden and a risk of malnutrition devoid of health benefits for this subgroup of patients.

First of all, it must be pointed out that individuals with potential CD may not always be completely asymptomatic and a GFD could be of benefit even in the absence of histologically evident mucosal changes [51,52]. Moreover, the adequacy of histology sampling has to be reviewed before classifying a patient with positive serology as potential CD, since at least four duodenal biopsies (according to some guidelines even six biopsies) are required to ensure an adequate diagnosis and some subjects may show histological changes only in the duodenal bulb [2,53,54]. It is a matter of debate if a reference pathologist should be consulted in contradictory cases. This claim is substantiated by data revealing a high interobserver variability in histopathology [55,56,57].

Kurppa et al., studied the effects of a GFD in screening-detected asymptomatic patients with CD [51]. They included also 40 patients positive to IgA-anti-endomysium antibodies that were at risk for CD (potential CD), who were randomized to either group A following a GFD or group B continuing a normal diet for a year. Despite the fact that all subjects prior to the study described themselves as “asymptomatic”, only group A improved significantly on the Gastrointestinal Symptoms Rating Scale. However, social functioning was impaired by the GFD. The authors concluded that even apparently asymptomatic patients with positive antibodies benefit from a GFD.

In another study, Mandile et al., evaluated the effect of GFD on clinical symptoms and mucosal histology in children with potential CD [58]. After one year on GFD no significant differences were observed in terms of Marsh grade, lamina propria CD25+ cells, CD3+ and γδ+ intraepithelial lymphocytes density and intestinal anti-TG2 deposits. However, about half of the patients reported improved clinical symptoms. A similar result was observed by Volta et al. [52]. Patients with potential CD that showed symptoms improved clinically by following a GFD.

**Figure 1:**
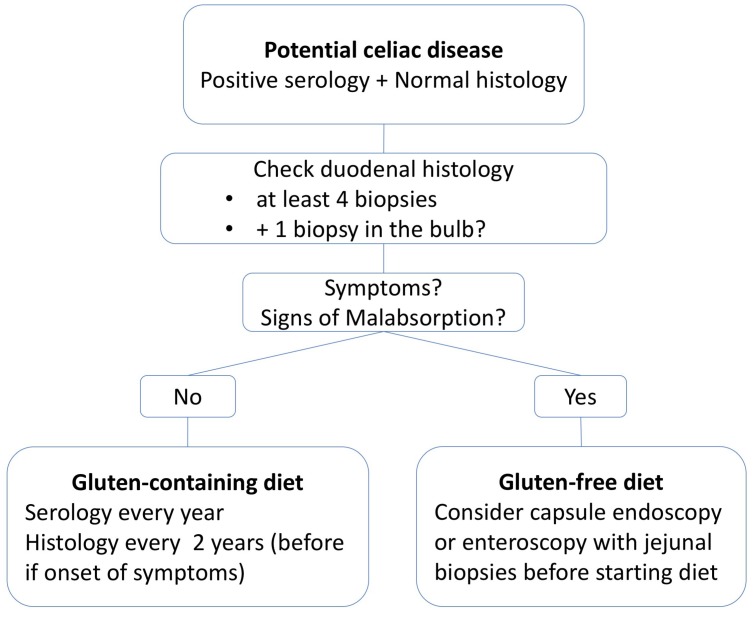
Rational approach to patients with potential CD.

On the other hand, it is interesting to assess the question how many patients with positive CD serology and normal histology will eventually develop CD characteristic celiac histology. Biagi et al., found only 35% to have developed a flat mucosa in the course of their disease. However, 29% of those with a normal histology decided to switch onto a GFD early on in the course, making it impossible to judge on their further natural course [59]. Interestingly, a reversal of CD serology has also been observed, especially in children [60]. Auricchio et al., studied 210 children with potential CD of which 175 were left on a gluten-containing diet [61]. Antibodies and clinical symptoms were checked twice a year and a small bowel biopsy was taken every second year. They found that 37% of the subjects showed fluctuation and 20% normalization of antibody production over the years, most of them (67%) without ever displaying mucosal damage during 9 years of follow-up. They concluded that a GFD might not always be necessary for individuals with potential CD. Volta et al., after collecting data from 77 adult patients from CD, proposed to prescribe a GFD only to clinically symptomatic patients with potential CD due to the observed improvement of symptoms after gluten withdrawal [52]. In asymptomatic patients who remained on a gluten-containing diet, progression to overt CD could merely be observed in 1 of 16 patients during a 5-years follow up. A possible rational approach in view of available data is shown in Figure 1.

## 6. Is There A Way Towards Gluten Reintroduction in Celiac Disease?

In recent years, there has been active debate on whether the GFD should be continued lifelong in all celiac patients. Data on the natural history of CD suggest that an excess of mortality, possibly derived from lymphoproliferative disorders, may be subsequent to higher/longer exposure to dietary gluten [7,33,51,62]: On this basis, the GFD should be continued indefinitely not only to prevent clinical relapse and malabsorption, but also to prevent complications.

Nevertheless, there are a number of studies published during the last 30 years that showed proof for the existence of a “latent” form of CD, where symptoms and histologic changes disappear in the course of disease despite consumption of a GFD. As early as 1989, a Finnish study tried to evaluate the possibility of development of gluten tolerance in children: 38 post pubertal children with CD were rebiopsied before a gluten challenge was carried out [63]. Eleven percent did not relapse (clinically and histologically) after 2 years, indicating a possible recovery from CD in this small subgroup.

Matysiak-Budnik et al., in 2007 conducted a retrospective analysis of clinical symptoms, mucosal recovery and laboratory findings of patients diagnosed with CD in childhood who, despite diagnosis, did not follow a GFD but resumed a normal diet and remained clinically silent [64]. They found that about a fifth of CD patients developed long-term latency (normal duodenal histology) after gluten reintroduction. Patients who remained clinically silent but displayed histological abnormalities were at increased risk for osteoporosis. The authors therefore concluded that a GFD is advisable in patients with asymptomatic CD, but that a subgroup of celiac patients may actually become gluten tolerant over time. However, tolerance could be transient and therefore demands regular follow-up. In fact, two “gluten-tolerant” patients actually showed clinical and mucosal relapse during the follow up.

Hopman et al., performed a follow-up with 77 patients who had been diagnosed with CD for over ten years [65]. Gluten consumption, symptoms, bone mineral density and antibodies were examined. Two thirds adhered to GFD, 15% were partially compliant and 23% followed a regular gluten-containing diet. Interestingly, biopsies revealed a normal mucosal histology in four of eight patients on gluten-containing diet and in all patients who were partially compliant. The authors concluded that development of tolerance to gluten was possible in some patients with CD. They suggest a regular follow-up to determine if this tolerance is permanent or not.

Although poor adherence to the GFD is the major predictor of persistence of mucosal lesions at follow-up histology, a recent study by Norsa et al., showed no excess in mortality among celiac patients with a long history of CD with poor or no adherence to the GFD, also reporting a proportion of patients of almost 30% with no relapse of villous atrophy despite chronic voluntary gluten ingestion [8].

### Who Could Benefit Most from Gluten Reintroduction?

Screening-detected CD patients are a subgroup of patients that experience more difficulty accepting the diagnosis and permanent dietary restriction since its justification, in the absence of symptoms, is less evident than in symptomatic patients that experience immediate improvement in health when adhering to GFD [66]. They report a decreased perception of health on a GFD [6] and may feel less motivated to adhere to the diet than symptomatic patients [67]. Accordingly, a recent study published in August 2018 studied the long-term health and treatment outcomes in screening-detected CD patients [66]. At a median of 18.5 years after diagnosis, 236 patients completed follow-up questionnaires. With regard to clinical symptoms and quality of life, the screening-detected subgroup showed more anxiety and lower general well-being than CD patients that had been diagnosed due to clinical suspicion. In this group of patients, the GFD is recommended in order to avoid long-term consequences of malabsorption. Moreover, epidemiological data on the excess mortality of CD complications linked to gluten exposure have been questioned [8,48].

In other words, considering the reported proportion—20–30% of CD patients able to develop a gluten tolerance over time and the limited risk of developing osteoporosis and complications over a short time of histological relapse of villous atrophy—it needs to be questioned whether a gluten reintroduction (or “re-challenge”) under careful follow-up could be a rational therapeutic option in patients with asymptomatic celiac disease. As follow-up, lifelong assessments including histology would be needed, in order to detect a possible relapse and worsening of mucosal histology. More research is needed before this option can be implemented into the clinical routine, however.

## 7. Conclusions and Future Perspectives

In patients affected by CD, the GFD ensures improvement of clinical symptoms and signs of malabsorption in the vast majority of cases. Despite extensive research aimed at developing alternative therapies for CD, the GFD remains the only effective treatment available to date [68]. However, it is not easy to follow and may result in a psychological and social burden for patients [5,6]. For this reason, the GFD should be prescribed only once CD diagnosis has been established by means of serology and duodenal histology.

In potential CD, the diet should be reserved to subjects reporting symptoms, while those asymptomatic can be maintained on a gluten-containing diet but should be followed up on a regular basis.

In patients with symptomatic CD, the diet should be followed strictly in view of the risk for complications such as osteoporosis and other consequences of malabsorption. Gluten exposure neither appears to be linked to the onset of other autoimmune disorders nor has an increased risk for malignancies been shown in asymptomatic CD so far. The current literature available, however, does not provide enough evidence to safely recommend a gluten-containing regimen in asymptomatic CD. More research on this topic is needed before introducing a gluten re-challenge for asymptomatic CD patients into the clinical routine.

The natural history of CD on a gluten-containing diet is still far from understood: It is unclear whether the large group of patients with mild or asymptomatic CD is indeed at risk of developing long-term-complications such as EATL. Further studies aimed at investigating the effects of gluten reintroduction in CD are required to identify the subgroup of patients that may develop gluten tolerance over time, without increasing the risk for CD related complications.

## Figures and Tables

**Table 1:** The spectrum of clinical presentations of celiac disease.

Clinical Features	Celiac Disease			
	**Classical**	**Non-Classical**	**Asymptomatic**	**Potential**
Malabsorption syndrome diarrhea, steatorrhea, weight loss/growth failure, with or without anemia	+	−	−	−
Gastrointestinal symptoms abdominal pain, bloating, constipation	+/−	+/−	−	−
Extraintestinal manifestations fatigue, osteopenia/osteoporosis, iron deficiency, neurological disorders	+/−	+/−	−	−
Serology (tTG/EMA)	+	+	+	+
Histological alterations (Marsh)	+	+	+	−

tTG, anti-transglutaminase antibodies; EMA, anti-endomysial antibodies.

**Table 2:** Relevant original studies evaluating the safe daily gluten threshold for celiac disease patients (adapted from Akobeng et al. [17]). CD: celiac disease; GFD: gluten-free diet; IEL: intraepithelial lymphocytosis; RCT: Randomized Controlled Trial.

Study	Methods	Participants	Diet	Aim of the Study	Results/Conclusion
Catassi 2007 [9]	RCT from Italy	39 adults with CD on GFD for >2 years	90 days, - 50 mg gluten/day (*n* = 13) - 10 mg gluten/day (*n* = 13) - 50 mg placebo/day (*n* = 13)	To evaluate the safe threshold of ingestion of contaminating gluten	Ingestion of contaminating gluten should be lower than 50 mg/day
Collin 2004 [10]	Cross-sectional study from Finland	76 adults, 16 children with CD on GFD for 1–10 years	Median 2 years - naturally GFD (*n* = 28) - wheat starch-based GFD (*n* = 64)	To establish a limit for residual gluten in gluten-free products	Threshold should be lower than 100 mg/day
Peräaho 2003 [18]	RCT from Finland	57 adults with newly diagnosed, untreated CD	1 year - wheat-starch-based-GFD (*n* = 28) - natural GFD (*n* = 29)	To evaluate the difference between a wheat starch-based GFD and natural GFD	No histological/clinical differences between the two groups
Lohiniemi 2000 [19]	Cross-sectional study from Finland	58 adults with CD, 110 healthy controls	Patients on wheat starch-based GFD	To evaluate the effect of wheat-starch based GFD on clinical symptoms	Wheat starch-based gluten-free products are suitable for CD patients
Selby 1999 [20]	Cross-sectional study from Australia	89 adults with CD	- Codex GFD (<500 ppm) (*n* = 39) - GFD < 30 mg (*n* = 50)	To evaluate the effect of gluten traces in GFD	No histological differences between the groups
Catassi 1993 [14]	Randomized controlled trial from Italy	20 children with CD on GFD	4 weeks - 100 mg gliadin/day ≈ 200 mg gluten/day (*n* = 10) - 500 mg gliadin/day ≈ 1 g gluten/day (*n* = 10)	To evaluate the effects of chronic ingestion of gliadin	Histological deterioration and increase in IEL count in both groups, clinical relapse in 30% of 1 g-group
